# Kawasaki disease in France, Kawanet: incomplete forms are frequent and associated with a high frequency of cardiac complications

**DOI:** 10.1186/1546-0096-12-S1-P124

**Published:** 2014-09-17

**Authors:** I Koné-Paut, M Darce Bello, E Merlin, E Launay, A Faye, F Boralevi, S Di Filippo, E Bosdure, JB Armengaud, S Tellier, A Arnoux, R Cimaz, M Piram

**Affiliations:** 1 Pediatrics, CHU Bicêtre, Le Kremlin Bicêtre, France; 2 Pediatrics, CHU Clermont-Ferrand, Clermont-Ferrand, France; 3 Pediatrics, CHU Nantes, Nantes, France; 4 Pediatrics, CHU Robert Debré, Paris, France; 5 Dermatology, CHU Bordeaux, Bordeaux, France; 6 Cardiology, HC Lyon, Lyon, France; 7 Pediatrics, CHU la Timone, Marseille, France; 8 Pediatrics, CHU Trousseau, Paris, France; 9 Pediatrics, CHU Purpan, Toulouse, France; 10 Clinical Research, CHU Bicêtre, Le Kremlin Bicêtre, France; 11 Pediatric Rheumatology, A. Meyer Institut, Firenze, Italy; 12 Pediatric Rheumatology, CHU Bicêtre, Le Kremlin Bicêtre, France

## Introduction

KD is the main vasculitis affecting children before 5 years and the leading cause of acquired heart disease. The epidemiologic of KD is few reported in France within a population from different ethnic backgrounds. Even IVIG is still the standard treatment; the management of patients at risk for cardiac complications may change toward reinforced (and new) therapeutic approaches.

## Objectives

Kawanet is a clinical and biological data repository aimed to define the epidemiological characteristics of KD in France. Kawanet will compare clinical characteristics between distinct ethnic backgrounds and will define risk factors for resistance to standard treatment (IVIG) and for cardiac complications.

## Methods

Targeted institutional physicians received information on a national registry for KD. All patients suspected with KD and seen since January 2011 were eligible to enter the study. An eCRF was implemented in a web database. IRB approval for data storage were obtained.The included patients without the AHA international criteria were reviewed by an experts' committee.

## Results

468 cases were entered by 84 physicians from 65 centers. The AHA classification gave: 280 complete (≥ 4 criteria), and 73 incomplete (≤ 3 criteria with coronary dilatation). An expert consensus classified 48 other patients (≤ 3 criteria but agreement for IVIG treatment) leading 401 patients considered as KD (M229/F172). 45 patients were doubtful and 22 not classified for incomplete data. The median age at diagnosis for the Kawasaki was 3.1y (2m-14y). Their ethnical backgrounds were: European Caucasian 67%, Eastern Caucasian or North African 15% afro-Caribbean, 13%, Asian 4% and mixed ancestry 1%. The clinical symptoms were (%): conjunctivitis 84, cheilitis 82, diffuse exanthema 74, modification of the extremities 73, oral erythema 66, cervical adenopathy 52, raspberry tongue 49, seat erythema 26, perineal desquamation 18 and BCG erythema 5. The cardiac complications were: coronary dilatation 30%, pericarditis 15%, coronary aneurysm 4%, and myocarditis 3% (1 death). 392/401 (98%) patients received IVIG, 21% (n=64) required 2 courses and 5 patients 3 courses. 11% required steroids, 93% received Aspirin and 1 Anti-TNF. The mean delay between fever onset and treatment was 6 days. The factors associated with the coronary abnormalities were: male gender (p=0.01), young age KD onset (p=0.03) and resistance to IVIG (p=0.03).

## Conclusion

KD diagnosis remains challenging and overdiagnosis represents at least 10% of cases in this registry. Incomplete forms of KD account for 37 % and are associated with coronary dilatation/aneurysm (34%; p<0.01) and a high rate of IVIG resistance. Unlike previous studies, our population is very mixed with 28 % of children from the Middle East and Africa, in whom KD is still few reported.This study is supported by grants form APHP: PHRC2009, LFB and private (patient/family) donation

## Disclosure of interest

None declared.

